# Disparities exist among US adolescents in the receipt of transition to adult healthcare services: the differential impact of social determinants of health, healthcare needs, and COVID-19

**DOI:** 10.3389/fpubh.2024.1452418

**Published:** 2024-12-18

**Authors:** Tyra C. Girdwood, Susan G. Silva, Gary R. Maslow, Sharron L. Docherty

**Affiliations:** ^1^School of Nursing, Duke University, Durham, NC, United States; ^2^School of Medicine, Duke University, Durham, NC, United States

**Keywords:** adolescents, special healthcare needs, transition to adult care, health inequities, COVID-19, social determinants of health

## Abstract

**Introduction:**

We examined the influence of special healthcare needs, onset of the COVID-19 pandemic, and their interaction on receiving transition services to prepare for future adult care among US adolescents, and whether social determinants of health moderated the relationship of these factors with receiving transition services.

**Methods:**

We analyzed the National Survey of Children's Health (2019, 2020–2021) using adjusted multivariable logistic regression models. We assessed a repeated cross-sectional, nationally representative sample of adolescents aged 12–17 years old. Sampling weights were used to generalize samples to the populations of interest. The main outcome was receipt of transition services to prepare for future adult healthcare. Measures included pre vs. post COVID-19 onset, special healthcare needs, and social determinants of health (health insurance, food sufficiency, neighborhood safety, household language, race/ethnicity, and household poverty level). Sex and two-parent households were included as covariates.

**Results:**

A total of 45,935 adolescents were included, with *N*=12,230 in the pre COVID-19 group and *N*=33,705 in the post COVID-19 group. We found statistically significant higher odds of receiving transition services among adolescents with special healthcare needs (*95 CI* = 1.23, 1.58), females (*95 CI* = 1.09, 1.39), and during pre COVID-19 (*95 CI* = 1.14, 1.45). Private insurance (*95 CI* = 1.03, 1.37), English as primary household language (*95 CI* = 1.19, 2.27), and race/ethnicity were significant predictors of receipt of transition services. Neighborhood safety significantly moderated (*95 CI* = 1.70, 6.60) the relationship between special healthcare needs and receipt of transition services.

**Discussion:**

This population-based study identified significant disparities in receipt of transition services provided to US adolescents via the differential impact of social determinants of health, special healthcare needs, and COVID-19 onset on receipt of services.

## 1 Introduction

Despite national priorities to support adolescents transitioning from pediatric to adult healthcare, US-wide surveys continue to document low rates of adolescents receiving transition services that could prepare them to thrive in adult-focused environments (1). Providing all adolescents services that develop their health literacy, self-care skills, and smooth the transition process is necessary as they develop and experience changes in healthcare status, care providers, work, school, and lifestyles (2). Prior to 2020, transition programs for adolescents with or without special healthcare needs (SHCN) were not widely available outside of children's hospitals (3). The disruption of the COVID-19 pandemic onset in 2020 created additional challenges in providing transition services (4, 5). Most recently, an estimated 11.8 million US adolescents experienced missed or delayed care visits in 2022 due to COVID, indicating potential gaps in care coordination (1).

Gaps in receiving services to prepare for transition are also influenced by social determinants of health (SDOH) like household poverty level, race and ethnicity, food insufficiency, and unsafe neighborhoods (6, 7). The consequences of inadequate transition preparation include increased risk for mental health comorbidities, low medication adherence, and poor health outcomes (2, 8, 9). This may be especially detrimental to adolescents with SHCN like sickle cell disease or cystic fibrosis for whom the transition is made more challenging due to complex disease management and treatment regimens (3, 9, 10). Notably, unnecessary admissions, complications, and readmissions resulting from failed care coordination/transitional care cost the U.S. healthcare system an estimated $27.2–78.2 billion dollars annually (11).

Researchers have identified the COVID-19 pandemic and SDOH as key forces impacting the health of transition-age adolescents in the US (4–7, 12). COVID-19 has impacted socio-economic opportunities for families, worsened social determinants of health, and created delays in transitions to adult healthcare (4, 5, 12). Additionally, adolescent females and adolescents residing in two-parent households have higher rates of receiving transition services/preparation compared to their peers (13). Studies suggest adolescents without SHCN receive less transition-related information from providers than adolescents with SHCN (5). However, while researchers have investigated the associations between person- and family-centered factors and the receipt of transitional care, there is a gap in exploring associations between systemic factors (i.e., COVID-19 pandemic) and multi-level SDOH factors on receipt of transition services (6, 7, 13, 14). This gap makes it difficult to understand health inequities inherent in receipt of transitional care provided to diverse adolescents in the US (6, 7, 13, 14).

Thus, our study goal was to determine whether US adolescents with SHCN were less likely to receive transition services to prepare for future adult healthcare compared to their peers, and to evaluate the differential impact of COVID-19 onset on receipt of these services among those with special healthcare needs and those without. Additionally, we examined SDOH that modified the effect of SHCN and COVID-19 on receipt of transition services. Hence, we aimed to determine the influence of SHCN, onset of COVID-19, and their interaction on the receipt of transition services, covarying for adolescent sex and two-parent household (13). Further, we aimed to identify SDOH that moderated the impact of these two factors (SHCN and/or COVID-19) on receipt of services, covarying for adolescent sex and two-parent household.

## 2 Methods

### 2.1 Design

We used a cross-sectional 2 × 2 factorial design to explore data from the 2019 and the combined 2020 and 2021 National Survey of Children's Health (NSCH), which enrolls a repeated cross-sectional nationally representative sample of youth aged 0–17 years old in the U.S. each year (15–17). The data were collected from independent samples, with 2019 data collected prior to the COVID-19 pandemic onset and 2020–2021 data obtained after COVID-19 onset. The Duke University Institutional Review Board determined this study as exempt as data were anonymized and publicly available. Data were reported using the STROBE statement (see Supplementary Table 2) (18).

### 2.2 Data source

The NSCH is a publicly available, fully-deidentified database with data collected each enrollment year from caregivers via an online or paper survey about one randomly selected child in their household (15). The NSCH survey included items on healthcare, individual and family characteristics, and school and community factors (19–21). Survey data elements were collected from June 2019 to January 2020 for the 2019 dataset (weighted survey response rate: 42.4%) (19), July 2020 to January 2021 for the 2020 dataset (weighted survey response rate: 42.4%) (20), and June 2021 to January 2022 for the 2021 dataset (weighted survey response rate: 40.3%) (21).

### 2.3 Participants

**Figure 1:**
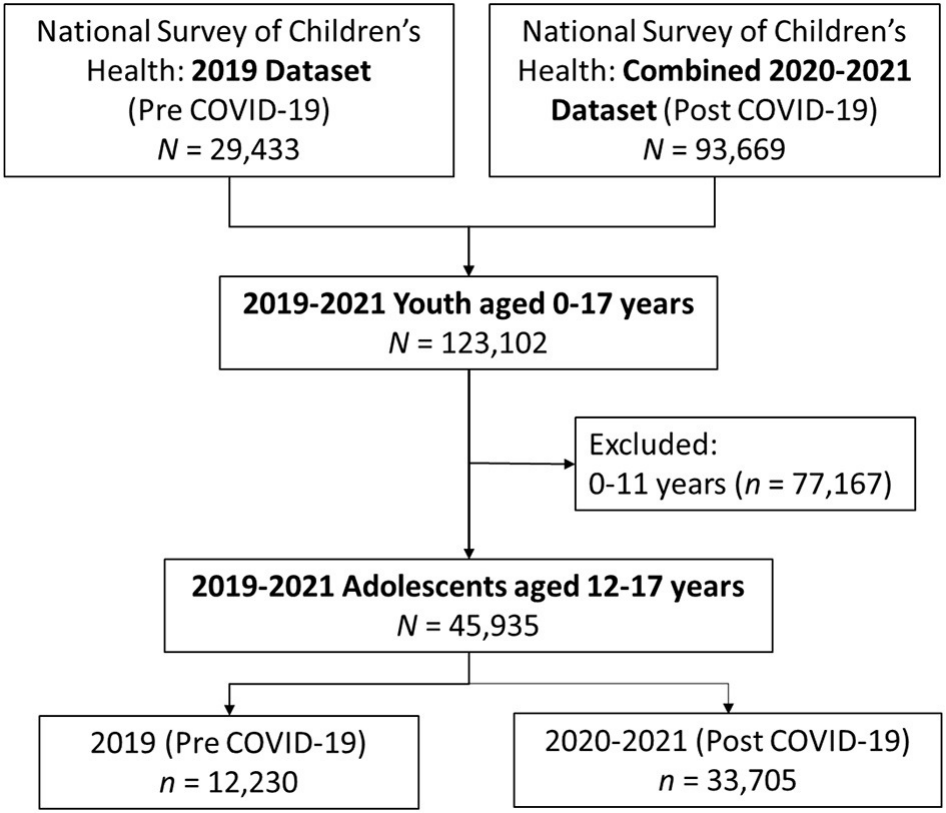
Flow diagram of the final analysis sample.

For this study, we included only data on transition-age adolescents (12–17 years) with and without special healthcare needs. The final analysis sample was 45,935 adolescents, with *N* = 12,230 in the pre COVID-19 group and *N* = 33,705 in the post COVID-19 group (see Figure 1).

### 2.4 Measures

Key analytic measures, definitions, and coding for the final analyses are summarized in Supplementary Table 1.

#### 2.4.1 Main outcome

The outcome was receipt of preparation services for transition to adult healthcare, coded as no (0) or yes (1). Hereafter referred to as “transition services”, this outcome was defined as having received early development of self-care skills, one-on-one provider communication skills, and information on the transition process and changes in healthcare. This outcome was derived in the dataset from three components: (1) doctor spoke with the adolescent privately without an adult in the room during the last medical care visit, (2) a discussion about transitioning to adult care occurred, and (3) doctors actively worked with the adolescent to gain skills and understand changes in healthcare (16, 17). If there was a yes response to any of the three items, then the adolescent was coded as having received transition services.

#### 2.4.2 Special healthcare needs

Special healthcare needs of adolescents (SHCN vs. no SHCN) was operationalized by the Maternal and Child Health Bureau's consequences-based definition of children, with SHCN defined as an adolescent experiences a medical or health condition lasting 12 months or more that requires prescription medication, or above average use of medical/mental/educational services, or has functional limitations, or requires utilization of specialized therapies, or incurs treatment for emotional or developmental problems (16, 17). We chose a binary assessment of special healthcare needs because despite complexity of care, adolescents with any special healthcare needs utilize a larger portion of healthcare and financial resources, they require services beyond what is generally required by adolescents, and they require care coordination among primary care, medical specialty, and/or nonmedical specialty providers compared to adolescents with no special healthcare needs (22).

#### 2.4.3 COVID-19 onset group

Adolescents were divided into two groups based on whether the data were collected before (year 2019) or after (years 2020–2021) the onset of the COVID-19 pandemic (pre- vs. post-COVID).

#### 2.4.4 Social determinants of health

Seven social determinants of health were considered including private health insurance (no, yes); public health insurance (no, yes); food sufficiency (sometimes/often not afford enough to eat, always could afford enough to eat but not always nutritious meals, always afford to eat nutritious meals); safe neighborhood (definitely agree, somewhat agree, somewhat/definitely disagree); household language (English, not English); race and ethnicity (Hispanic individuals, White non-Hispanic, Black non-Hispanic, Asian non-Hispanic, Other/multi-racial non-Hispanic); and household poverty level (no, yes—defined as 0–99% federal poverty level, FPL).

#### 2.4.5 Covariates

Sex (male, female) and two-parent household (no, yes) were included as covariates based on prior literature (13). Two-parent household was defined as two parents (married or not) who lived in the same household. The dataset only included adolescent age groups (i.e., 0–5 years, 6–11 years, 12–17 years) and not individual ages and thus age was not included as a covariate (16, 17).

#### 2.4.6 Design variables

The NCSH datasets provided survey design variables to obtain better parameter estimates, particularly standard error. The variables account for the complex survey design and adjust for under- or over-representation of subpopulations. Design variables applied were individual sampling weights (FWC), two strata variables (FIPSST, state of residence; STRATUM, identified households with children), and a cluster variable (HHID, unique household identifier) (16, 17).

### 2.5 Data analysis

Descriptive statistics were used to detail sample characteristics and key analytic variables. Non-directional statistical tests were performed with statistical significance set at 0.05. Effect sizes and their 95% confidence intervals were used to address clinical significance. Using SAS 9.4 software (Cary, NC) (23), data were analyzed with procedures designed for population-based research, thus, weighted analyses incorporated sampling weights and other design variables.

#### 2.5.1 Sample characteristics

The sample characteristics for the two COVID-19 groups were described and compared using Rao-Scott chi-square tests. Characteristics included SDOH, covariates, SHCN, and transition services.

#### 2.5.2 Transition services: role of COVID-19 onset and special healthcare needs

Covariate-adjusted multivariable logistic regression was used to examine the effects of COVID-19 group and SHCN and their interaction on transition services, after covarying for sex and two-parent household. The event of interest was receipt of transition services (1, yes). In the event of a significant interaction at the 0.05 level, *a posteriori* subgroup comparisons were planned to further delineate the interaction. Adjusted odds ratios (aORs) and 95% confidence intervals (CIs) were used to evaluate clinical significance (24).

#### 2.5.3 Transition services: moderating effects of social determinants of health

Seven SDOH were examined to determine whether the characteristic was either a non-specific predictor or moderator of the relationship between COVID-19 group and/or SHCN with transition services, after covarying for sex and two-parent household. Each SDOH was evaluated in separate multivariable logistic regression models in which the SDOH of interest and SDOH interactions were added to the core model described above. Each model included the following explanatory variables: (a) COVID-19 group and SHCN factors and their interaction; (b) sex and two-parent household as covariates; and (c) SDOH and its two-way and three-way interaction with the factors. If the COVID-by-SHCN interaction was not statistically significant at the 0.05 level, this interaction term and COVID-by-SHCN-by-SDOH interaction were both dropped from the final pragmatic moderator model. The SDOH was determined to be a non-specific predictor of transition services if only the main effect was statistically significant. The SDOH was determined to be a moderator when a two-way SDOH interaction with COVID and/or SHCN or a three-way interaction with COVID and SHCN was statistically significant.

### 2.6 Statistical power

The expected sample sizes for adolescents pre vs. post COVID-19 and SHCN vs. no SHCN provided at least 80% statistical power to test for main and interaction effects on transition services in a multivariable logistic regression model, covarying for SDOH and covariate terms and assuming two-tailed tests with significance set at 0.05 and small effect sizes (when *aOR* > 1: small effect = 1.50 or when *aOR* < 1: small effect = 0.67).

## 3 Results

### 3.1 Sample characteristics

As described in Table 1, when compared to the post COVID-19 group, the pre COVID-19 group had a significantly higher proportion of adolescents whose caregiver reported English as the primary household language (86% vs. 84%, *p* = 0.0228), a two-parent household (71% vs. 68%, *p* = 0.0503), and caregivers that somewhat or definitely disagreed to living in a safe neighborhood (unsafe neighborhood, 6% vs. 5%, *p* = 0.0069). Although statistically significant, the effect sizes were small. The proportion of adolescents with SHCN did not differ for the pre and post COVID-19 groups (26% vs. 25%, *p* = 0.6759).

**Table 1:** Characteristics of the COVID-19 onset groups.

**Characteristic**	**Pre-COVID group (*****N*** = **12,230)**				**Post-COVID group (*****N*** = **33,705)**				***p*-value**
	**Unweighted**		**Weighted**		**Unweighted**		**Weighted**		
	***f***	***%***	***%***	***95% CI***	***f***	***%***	***%***	***95% CI***	
**Race and ethnicity**									0.7029
Hispanic individuals	1,396	11.4	26.2	23.9–28.5	4,661	13.8	27.5	26.1–28.9	
White individuals NH	8,619	70.5	49.9	47.9–51.9	22,236	66.0	48.6	47.4–49.8	
Black individuals NH	797	6.5	13.8	12.3–15.3	2,447	7.3	14.1	13.2–15.0	
Asian individuals NH	616	5.0	4.7	4.0–5.4	1,896	5.6	4.4	4.0–4.8	
Other individuals NH	802	6.6	5.4	4.7–6.1	2,465	7.3	5.5	5.0–5.9	
**Public Insurance**	2,608	21.7	31.7	29.6–33.7	8,098	24.5	33.3	32.0–34.6	0.1850
**Private insurance**	9,217	76.8	65.0	62.9–67.1	24,504	74.2	62.7	61.4–64.0	0.0709
**Food sufficiency**									0.3087
Always afford nutritious meals	8,788	73.1	67.8	65.9–69.8	24,450	74.5	69.5	68.3–70.7	
Afford but not always nutritious	2,785	23.2	26.7	24.9–28.6	7,276	22.1	25.7	24.6–26.8	
Sometimes/often cannot afford	445	3.7	5.4	4.4–6.4	1,117	3.4	4.8	4.2–5.4	
**Safe neighborhood**									0.0069
Definitely agree	8,492	71.1	63.8	61.7–65.8	23,652	72.3	67.9	66.6–69.1	
Somewhat agree	3,083	25.8	30.4	28.4–32.4	8,056	24.6	27.4	26.2–28.6	
Somewhat/definitely disagree	367	3.1	5.9	4.6–7.2	1022	3.1	4.7	4.0–5.4	
**Household language**									0.0228
English	11,498	94.5	86.4	84.6–88.3	31,053	92.6	83.7	82.4–85.0	
Not English	674	5.5	13.6	11.7–15.4	2,479	7.4	16.3	15.0–17.6	
**Household poverty level**									0.3005
0-99% FPL	1,260	10.3	17.5	15.8–19.2	4,273	12.7	18.6	17.5–19.7	
100-400% FPL or greater	10,970	89.7	82.5	80.8–84.2	29,432	87.3	81.4	80.3–82.5	
**Special healthcare needs**									0.6759
Yes SHCN	3,630	29.7	25.4	23.7–27.1	10,458	31.0	25.8	24.8–26.8	
No SHCN	8,600	70.3	74.6	72.9–76.3	23,247	69.0	74.2	73.2–75.2	
**Sex**									0.8396
Male	6,320	51.7	51.2	49.2–53.3	17,545	52.1	51.0	49.7–52.2	
Female	5,910	48.3	48.8	46.7–50.8	16,160	48.0	49.0	47.8–50.3	
**Two-parent household**									0.0503
Yes	9,073	75.6	70.7	68.7–72.6	23,463	71.5	68.3	67.1–69.5	
No	2,928	24.4	29.3	27.4–31.3	9,368	28.5	31.7	30.5–32.9	
**Transition services**									0.0002
Yes, received	2,967	24.4	20.5	18.9–22.1	7,001	20.9	17.2	16.3–18.0	
No, did not receive	9,213	75.6	79.5	77.9–81.1	26,575	79.2	82.8	82.0–83.7	

95% CI, 95% Confidence Interval of the Percent; NH, Non-Hispanic; FPL, Federal Poverty Level; SHCN, Special Healthcare Needs; p-value from Rao-Scott Chi-square test using design variables (sampling weights, cluster, strata) to compare pre and post COVID-19 group differences in characteristics.

### 3.2 Transition services: role of COVID-19 onset and special healthcare needs

Only 21.7% (*n* = 9,968) of the total sample of adolescents received transition services. Further, the proportion of adolescents who had received transition services was significantly higher in the pre COVID-19 group compared to the post COVID-19 group (21% vs. 17%, *p* = 0.0002, Table 1). The covariate-adjusted multivariable logistic regression results in Table 2 indicated a significant main effect of COVID-19 group (*p* < 0.0001) and SHCN (*p* < 0.0001), but no interaction effect (*p* = 0.6492) on transition services, after covarying for sex and two-parent household. Specifically, the odds of receiving transition service were 28% higher (*aOR* = 1.28) among the pre COVID-19 adolescents compared to those post COVID-19, and were 40% (*aOR* = 1.40) higher for those with SHCN relative to those with no SHCN. Although two-parent household was not related to transition services, female adolescents had significantly higher odds of receiving transition services than male adolescents (*aOR* = 1.23, *p* = 0.0006). Estimated effect sizes for the findings in Table 2 were small.

**Table 2:** Transition services: role of COVID-19 onset and special healthcare needs.

**Factors and covariates**	***aOR***	***aOR 95% CI***	***p*-value**
**COVID-19 onset group**			< 0.0001
Pre COVID-19	1.28	1.14, 1.45	
Post COVID-19 *(ref)*			
**Special Healthcare Needs (SHCN)**			< 0.0001
Yes	1.40	1.23, 1.58	
No *(ref)*			
**Group-by-SHCN interaction**			0.6492
**Sex**			0.0006
Female	1.23	1.09, 1.39	
Male *(ref)*			
**Two-parent household**			0.6716
Yes	0.97	0.84, 1.12	
No (*ref*)			

N = 44,685; Covariate-adjusted multivariable logistic regression result; design variables applied (sampling weights, strata, and cluster) applied; aOR, adjusted odds ratio; aOR > 1 effect size cutoffs: small = 1.50, medium = 2.00, large = 3.00.

### 3.3 Transition services: moderating effects of social determinants of health

The COVID-19-by-SHCN interaction was not statistically significant in the prior analysis; thus, this two-way and three-way interaction term were omitted from the moderator analyses. A separate covariate-adjusted logistic regression model was used to evaluate the moderating effect of each SDOH.

Table 3 presents the regression model and results for the significant SDOH. The odds of receiving transition services were significantly higher for those with private insurance compared to peers (main effect only, *p* = 0.0193), and for those in which the primary household language was English compared to another language (main effect only, *p* = 0.0027). English as primary household language had a medium effect size (*aOR* = 1.64). Race and ethnicity was also a significant predictor of transition services (main effect only, *p* = 0.0103), with the *a posteriori* pairwise contrast indicating significantly: (a) greater odds of transition services for Non-Hispanic Other/multiracial groups and Non-Hispanic Whites relative to Hispanics individuals (*aOR* = 1.40 and 1.34, both *p* < 0.03, respectively) and (b) lower odds of transition services in Non-Hispanic Asians relative to Non-Hispanic Other/multiracial groups and Non-Hispanic Whites (*aOR* = 0.65 and 0.67, both *p* < 0.044, respectively).

**Table 3:** Social determinants of health (SDOH): association with transition services.

***N***	**Factors, covariates, and SDOH**	**aOR**	**aOR 95% CI**
43,918	COVID-19 onset group (pre vs. post group)	1.26^***^	1.09, 1.45
	Special Healthcare Needs (SHCN, yes vs. no)	1.45^***^	1.25, 1.68
	Sex (female vs. male)	1.22^***^	1.09, 1.38
	Two-parent household (yes vs. no)	0.91	0.79, 1.05
	***Private Insurance (yes vs. no)***	1.19^**^	1.03, 1.37
	*Private Insurance-by-group (p > 0.05)*		
	*Private Insurance-by-SHCN (p > 0.05)*		
44,460	COVID-19 onset group (pre vs. post group)	1.37^*^	1.02, 1.82
	Special Healthcare Needs (SHCN, yes vs. no)	1.23	0.89, 1.69
	Sex (female vs. male)	1.24^***^	1.10, 1.39
	Two-parent household (yes vs. no)	0.97	0.84, 1.12
	***Household language (English vs. not english)***	1.64^**^	1.19, 2.27
	*Household language-by-group (p > 0.05)*		
	*Household language-by-SHCN (p > 0.05)*		
44,685	COVID-19 onset group (pre vs. post group)	1.32^***^	1.13, 1.55
	Special Healthcare Needs (SHCN, yes vs. no)	1.55^***^	1.27, 1.90
	Sex (female vs. male)	1.24^***^	1.10, 1.40
	Two-parent household (yes vs. no)	0.94	0.81, 1.09
	***Race and ethnicity***		
	Asian individuals NH vs. Black NH	0.77	0.52, 1.15
	Asian individuals NH vs. Other NH	0.65^*^	0.43, 0.97
	Asian individuals NH vs. White individuals NH	0.67^*^	0.47, 0.97
	Asian individuals NH vs. Hispanic individuals	0.90	0.60, 1.37
	Black individuals NH vs. Other NH	0.84	0.64, 1.09
	Black individuals NH vs. White individuals NH	0.87	0.72, 1.05
	Black individuals NH vs. Hispanic individuals	1.17	0.89, 1.53
	Other NH vs. White individuals NH	1.04	0.85, 1.29
	Other NH vs. Hispanic individuals	1.40^*^	1.05, 1.87
	White individuals NH vs. Hispanic individuals	1.34^**^	1.08, 1.66
	*Race and ethnicity-by-group (p > 0.05)*		
	*Race and ethnicity-by-SHCN (p > 0.05)*		
44,233	COVID-19 onset group (pre vs. post group)	1.26	0.99, 1.59
	Special healthcare needs (SHCN, yes vs. no)	1.81^***^	1.42, 2.32
	Sex (female vs. male)	1.26^***^	1.12, 1.43
	Two-parent household (yes vs. no)	0.92	0.80, 1.07
	***Safe neighborhood***		
	Definitely agree	1.87^***^	1.34, 2.60
	Somewhat agree	1.39	0.99, 1.95
	Somewhat/definitely disagree (*ref*)		
	*Safe neighborhood-by-group (p > 0.05)*		
	**Safe neighborhood-by-SHCN** ^*^		

N = Data available; Covariate-adjusted multivariable logistic regression result; design variables applied (sampling weights, strata, and cluster) applied; aOR, adjusted odds ratio; NH, non-Hispanic; SHCN, special Healthcare Needs; aOR > 1 effect size cutoffs: small = 1.50, medium = 2.00, large = 3.00; aOR < 1 effect size cutoffs: small = 0.67, medium = 0.50, large = 0.33. ^*^p ≤ 0.05, ^**^p ≤ 0.01, ^***^p ≤ 0.001. Interactions in bold were statistically significant, and any interactions that are italicized were not statistically significant.

**Figure 2:**
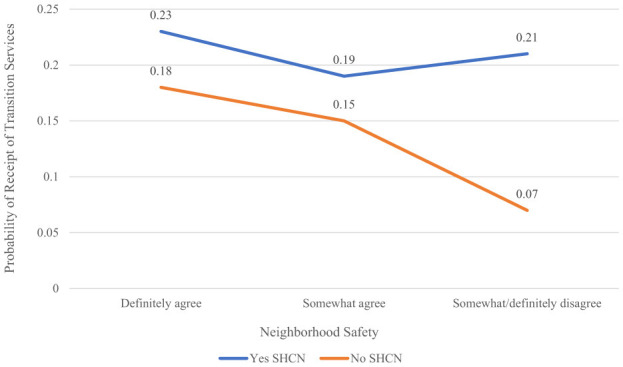
Safe neighborhood: moderator of SHCN and transition services relationship. SHCN, special healthcare needs.

Safe neighborhood was a moderator of the SHCN-transition services, as indicated by the significant safe neighborhood-by-SHCN interaction (*p* = 0.0318, Table 3). Figure 2 shows that adolescents with no SHCN had a significantly lower probability of transition services across all three levels of neighborhood safety compared to those with SHCN (SHCN main effect, *p* < 0.0001). However, probability of transition services for the two SHCN groups diverged when the neighborhood was reported to be unsafe. When safe neighborhood was rated as somewhat or definitely disagree, the probability of transition services was increased among those with SHCN and decreased among those without SHCN (*aOR* = 3.35, *95% CI* 1.70–6.60, *p* = 0.0005). Notably, this finding had a large effect size (*aOR* > 3.00).

## 4 Discussion

We aimed to examine the (1) impact of special healthcare needs, onset of COVID-19, and their interaction on receipt of transition services among US adolescents, and whether (2) social determinants of health moderated relationships between special healthcare needs and COVID-19 onset on receipt of transition services. We found significant disparities exist among US adolescents who receive transition services to prepare for adult-focused care.

In this nationally-representative sample of 45,935 adolescents, 78.3% did not receive transition services that could help them thrive in adult-focused care. This finding aligns with previously reported low rates of transition services provided to adolescents since 2016, however, we found an important distinction in care provided in relation to the COVID-19 pandemic (25). After the COVID-19 pandemic onset in 2020, the probability of receiving transition services was significantly lower for all adolescents compared to services provided in 2019, and significantly lower for males without SHCN compared to their peers. These results align with prior studies identifying healthcare barriers brought on by COVID-19 (e.g., fewer routine visits, delayed or altered care) which significantly disrupt the level of transitional care provided to adolescents (4, 12, 26). Our findings expand prior research by identifying that all adolescents in the post COVID-19 group suffered lower odds of receiving transition services compared to pre COVID-19, highlighting a critical need to adjust current, ineffective care practices. Notably, while all adolescents are disadvantaged from a systemic lack of adult-focused care preparation post COVID-19, adolescents living with chronic conditions like sickle cell disease and cystic fibrosis require more healthcare resources compared to their peers (22). The costs of a lack of adult-focused care preparation can be more than financial and contribute to the growing inequities that are experienced by these young adults and their families. These adolescents are at an increased risk for poor health and psychosocial outcomes if they lack preparation for self-care skills needed to manage their highly complex treatment regimens and care appointments in adulthood (10).

Private insurance (healthcare system-level factor), household language (family-level factor), and race and ethnicity (individual-level factor) were all statistically significant predictors of receiving transition services (27). Private insurance has been associated with increased transition readiness among adolescents with chronic conditions, indicating a need to enhance transitional services among adolescents with public insurance like Medicaid (13). Unlike previous literature where small samples limit analyses (13), our sample size allowed identification of the odds of receiving transition services as lower among adolescents who identified as non-Hispanic Asian, non-Hispanic Black, or Hispanic individuals and among those living in non-English speaking households. Additionally, it is important to note that English as primary household language had a medium effect size, suggesting clinical relevance and the need for more non-English resources and tools for families going through the transition process. Healthcare professionals in clinical settings must develop protocols to actively screen patients more at risk for not receiving transition services using tools like PRAPARE (28). The availability of culturally competent resources would assist in reducing health inequities that impact delivery of transitional care in these underserved populations (2). Also, the significant predictors that impact receipt of transition services do so via multiple levels (i.e., individual, family, healthcare system) (27). These results align with principles from the Healthcare Transition Research Consortium Model and SDOH frameworks (14, 27), and thus use of these frameworks in the development of transitional care interventions are more likely to target individual, family, and healthcare system-level factors that impact care delivery.

Adolescents with SHCN living in somewhat or definitely unsafe neighborhoods had a greater probability of receiving transition services compared to adolescents without SHCN. This finding had a large effect size, suggesting a high clinical relevancy and a critical need for mobile or community-based healthcare providers to assess neighborhood safety among transition-age adolescents. Families of adolescents without special healthcare needs who don't feel safe enough to venture outside of their homes may forgo healthcare services, indicating a significant subpopulation at risk for gaps in continuity of care. Adolescents in supportive neighborhoods were reported to experience positive transitional care services, like a provider describing the move to adult-focused care (7). Our results expand prior research by identifying neighborhood safety as a significant environment-level SDOH moderator that may not be actively assessed within inpatient or outpatient settings. Enhancing community-level partnerships and resources to reduce risks of harmful SDOH may help to improve transition service use outcomes among adolescents (2, 6, 7). For example, Medicaid managed care contracts could be used to evaluate for SDOH risks and pay for resources (29).

Our study was limited by the secondary analysis design, requiring use of previously specified variables. Thus, receipt of transition services may not capture the full scope of this construct (16, 17). In addition, data were caregiver-reported (i.e., missing the adolescent perspective), and asked caregivers to recall service use over the past year, increasing the risk for recall bias. The repeated cross-sectional nature of the survey design allowed for the determination of important associations, but future research using multi-level or structural equation modeling would allow testing for causal relationships. Future research could also assess complexity of healthcare needs as a subgroup of adolescents with special healthcare needs.

Recent social determinants of health frameworks suggest that strength-based factors, such as family resilience, may moderate SDOH and health inequities across the lifespan (14). Future studies using longitudinal data could assess the impact of SDOH factors on health outcomes for AYAs in young adulthood. Moreover, future health policy work should identify existing structures that could pay for community health workers and resources to reduce the impact of SDOH factors on receipt of transition services. For example, states can utilize Medicaid to address unmet needs contributed by social determinants of health, as is being done in North Carolina's Healthy Opportunities Pilots program (30). Since North Carolina has expanded Medicaid, the biggest enrollee group is young adults 19–29 years old, suggesting public health interventions via Medicaid would impact this transition-age population (31). Interventions that maximize care coordination and reduce disparities are estimated to save the US healthcare system $29.6–38.2 billion annually (11).

## 5 Conclusion

Inequities exist among US adolescents in the receipt of transition services to prepare for adult-focused care. Notably, post COVID-19, male adolescents without SHCN had the lowest probability of receiving transition services. Among adolescents who lived in unsafe neighborhoods, those without SHCN had lower odds of receiving transition services. These findings highlight the impact systemic issues have on transition services and the need for enhanced transitional care among these groups.

## Data Availability

Publicly available datasets were analyzed in this study. This data can be found here: https://www.childhealthdata.org/help/dataset.
