# Supplementary material for: Disparities exist among US adolescents in the receipt of transition to adult healthcare services: the differential impact of social determinants of health, healthcare needs, and COVID-19
Source: Front Public Health. 2024 Dec 18;12:1452418. doi: 10.3389/fpubh.2024.1452418 (PMC11691967; doi:10.3389/fpubh.2024.1452418)
Supplement: Supplementary file 1 [file Table_1.docx]

***Supplementary Material***

**1 eTable 1: Key Measures**

| **Variable** | **Variable Coding for Analysis** |
| --- | --- |
| COVID-19 onset group | Onset of COVID-19 pandemic group:  Pre COVID-19 (Year 2019)  Post COVID-19 (Years 2020-2021) |
| Special Healthcare Needs (SHCN) | Adolescent experiences a medical or health condition lasting 12 months or more that requires prescription medication, or above average use of medical/mental/educational services, or has functional limitations, or requires utilization of specialized therapies, or incurs treatment for emotional or developmental problems:  No, no SHCN  Yes, SHCN |
| Race and ethnicity | Adolescent’s race and ethnicity:  Hispanic  White, non-Hispanic (White NH)  Black, non-Hispanic (Black NH)  Asian, non-Hispanic (Asian NH)  Other/multi-racial, non-Hispanic (Other NH) |
| Private insurance | Adolescent has private health insurance:  No, public insurance or uninsured  Yes, private insurance or both public/private insurance |
| Public insurance | Adolescent has public health insurance:  No, private insurance or uninsured  Yes, public insurance or both public/private insurance |
| Food sufficiency | Food situation in the adolescent’s household in the past 12 months:  Sometimes/often could not afford enough to eat  Always could afford enough to eat but not always nutritious meals  Always afford to eat good nutritious meals |
| Safe neighborhood | Adolescent lives in a safe neighborhood:  Definitely agree that the neighborhood is safe  Somewhat agree that the neighborhood is safe  Somewhat/definitely disagree that the neighborhood is safe |
| Household language | Primary language spoken in the household:  English  Not English |
| Household poverty level | Household income based on imputed federal poverty level (FPL) status:  No, 100-400% FPL or greater  Yes, 0 to 99% FPL |
| Transition services | Outcome, receipt of preparation services for future transition to adult healthcare:  0 = No, adolescent did not receive necessary transition services  1 = Yes, adolescent received necessary transition services |
| Sex | Covariate, adolescent sex:  Male  Female |
| Two-parent household | Covariate, two-parent household:  No (single parent, grandparent, or other family type household)  Yes, two parents together who are married or not living in the same household |

**2 eTable 2: STROBE Statement for Cross-Sectional Studies**

|  | Item No | Recommendation | Page No |
| --- | --- | --- | --- |
| **Title and abstract** | 1 | (*a*) Indicate the study’s design with a commonly used term in the title or the abstract | 1 |
|  |  | (*b*) Provide in the abstract an informative and balanced summary of what was done and what was found | 1-2 |
| Introduction | | | |
| Background/rationale | 2 | Explain the scientific background and rationale for the investigation being reported | 2 |
| Objectives | 3 | State specific objectives, including any prespecified hypotheses | 3 |
| Methods | | | |
| Study design | 4 | Present key elements of study design early in the paper | 3-5 |
| Setting | 5 | Describe the setting, locations, and relevant dates, including periods of recruitment, exposure, follow-up, data collection | 3 |
| Participants | 6 | (*a*) Give the eligibility criteria, and the sources and methods of selection of participants | 3 |
| Variables | 7 | Clearly define all outcomes, exposures, predictors, potential confounders, and effect modifiers. Give diagnostic criteria, if applicable | 3-5 |
| Data sources/ measurement | 8 | For each variable of interest, give sources of data and details of methods of assessment (measurement). Describe comparability of assessment methods if there is more than one group | 3-5 |
| Bias | 9 | Describe any efforts to address potential sources of bias | 5 |
| Study size | 10 | Explain how the study size was arrived at | 5-6 |
| Quantitative variables | 11 | Explain how quantitative variables were handled in the analyses. If applicable, describe which groupings were chosen and why | 5 |
| Statistical methods | 12 | (*a*) Describe all statistical methods, including those used to control for confounding | 5 |
|  |  | (*b*) Describe any methods used to examine subgroups and interactions | 5 |
|  |  | (*c*) Explain how missing data were addressed | 5 |
|  |  | (*d*) If applicable, describe analytical methods taking account of sampling strategy | 5 |
|  |  | (*e*) Describe any sensitivity analyses | 5 |
| Item Recommendation Page No | | | |
| Results | | | |
| Participants | 13 | (a) Report numbers of individuals at each stage of study—eg numbers potentially eligible, examined for eligibility, confirmed eligible, included in the study, completing follow-up, and analyzed | 3;6 |
|  |  | (b) Give reasons for non-participation at each stage | 6 |
|  |  | (c) Consider use of a flow diagram | table1 |
| Descriptive data | 14 | (a) Give characteristics of study participants (eg demographic, clinical, social) and information on exposures and potential confounders | 6 |
|  |  | (b) Indicate number of participants with missing data for each variable of interest | See tables |
| Outcome data | 15 | Report numbers of outcome events or summary measures | 6 |
| Main results | 16 | (*a*) Give unadjusted estimates and, if applicable, confounder-adjusted estimates and their precision (eg, 95% confidence interval). Make clear which confounders were adjusted for and why they were included | 6-7 |
|  |  | (*b*) Report category boundaries when continuous variables were categorized | N/A |
|  |  | (*c*) If relevant, consider translating estimates of relative risk into absolute risk for a meaningful time period | N/A |
| Other analyses | 17 | Report other analyses done—eg analyses of subgroups and interactions, and sensitivity analyses | 6-7 |
| Discussion | | | |
| Key results | 18 | Summarize key results with reference to study objectives | 7-8 |
| Limitations | 19 | Discuss limitations of the study, taking into account sources of potential bias or imprecision. Discuss both direction and magnitude of any potential bias | 8 |
| Interpretation | 20 | Give a cautious overall interpretation of results considering objectives, limitations, multiplicity of analyses, results from similar studies, and other relevant evidence | 7-8 |
| Generalizability | 21 | Discuss generalizability (external validity) of the study results | 7-8 |
| Other information | | | |
| Funding | 22 | Give the source of funding and the role of the funders for the present study and, if applicable, for the original study on which the present article is based | N/A |
